# Influence of virtual reality soccer game on walking performance in robotic assisted gait training for children

**DOI:** 10.1186/1743-0003-7-15

**Published:** 2010-04-22

**Authors:** Karin Brütsch, Tabea Schuler, Alexander Koenig, Lukas Zimmerli, Susan Mérillat (-Koeneke), Lars Lünenburger, Robert Riener, Lutz Jäncke, Andreas Meyer-Heim

**Affiliations:** 1 Institute of Psychology, Division Neuropsychology, University of Zurich, Switzerland; 2 Institute of Human Movement Sciences, ETH Zurich, Switzerland; 3 Sensory-Motor Systems Lab, ETH Zurich, Switzerland; 4 Hocoma AG, Volketswil, Switzerland; 5 SCI Center, University Hospital Balgrist, Zurich, Switzerland; 6 Rehabilitation Center Affoltern a. A., University Children's Hospital Zurich, Switzerland

## Abstract

**Background:**

Virtual reality (VR) offers powerful therapy options within a functional, purposeful and motivating context. Several studies have shown that patients' motivation plays a crucial role in determining therapy outcome. However, few studies have demonstrated the potential of VR in pediatric rehabilitation. Therefore, we developed a VR-based soccer scenario, which provided interactive elements to engage patients during robotic assisted treadmill training (RAGT). The aim of this study was to compare the immediate effect of different supportive conditions (VR versus non-VR conditions) on motor output in patients and healthy control children during training with the driven gait orthosis Lokomat^®^.

**Methods:**

A total of 18 children (ten patients with different neurological gait disorders, eight healthy controls) took part in this study. They were instructed to walk on the Lokomat in four different, randomly-presented conditions: (1) walk normally without supporting assistance, (2) with therapists' instructions to promote active participation, (3) with VR as a motivating tool to walk actively and (4) with the VR tool combined with therapists' instructions. The Lokomat gait orthosis is equipped with sensors at hip and knee joint to measure man-machine interaction forces. Additionally, subjects' acceptance of the RAGT with VR was assessed using a questionnaire.

**Results:**

The mixed ANOVA revealed significant main effects for the factor CONDITIONS (p < 0.001) and a significant interaction CONDITIONS × GROUP (p = 0.01). Tests of between-subjects effects showed no significant main effect for the GROUP (p = 0.592). Active participation in patients and control children increased significantly when supported and motivated either by therapists' instructions or by a VR scenario compared with the baseline measurement "normal walking" (p < 0.001).

**Conclusions:**

The VR scenario used here induces an immediate effect on motor output to a similar degree as the effect resulting from verbal instructions by the therapists. Further research needs to focus on the implementation of interactive design elements, which keep motivation high across and beyond RAGT sessions, especially in pediatric rehabilitation.

## Background

Given the degree of walking impairments often caused by neurological disorders such as stroke, traumatic brain injury, spinal cord injury or cerebral palsy (CP), one major aim of rehabilitation is the restoration of such elementary capabilities. Regaining walking capacity was identified by stroke patients as one of the most important goals of rehabilitation [1-3]. In general, the recovery of motor functions after neural injury or disease depends on a variety of factors, including the nature and quantity of rehabilitation efforts [4,5]. However, conventional rehabilitative training programmes are often shorter and less intensive than required to obtain an optimal therapeutic outcome. Nor do they adequately increase the patients' motivation or promote their active participation. Several studies support the fact that patients' motivation plays a crucial role in determining therapy outcome and that, in certain patient populations it may even be the most critical factor in defining the success of the rehabilitation training (e.g. in stroke patients) [4,6-8]. Moreover, it has been suggested that a more challenging and competitive situation, as provided by virtual environments might increase patient's motivation to actively participate and thus shorten the time needed for motor skill recovery [4]. Furthermore, it is believed that passive guidance is less effective for motor learning and restoration of walking compared to active performance [9,10]. Preliminary results indicate that virtual reality (VR) offers powerful therapy options within a functional, purposeful and motivating context [11,12]. Previous studies, especially in pediatric rehabilitation, have demonstrated the potential of VR with regard to various aspects (e.g. improvements of life skills, mobility, cognitive abilities, fun and motivation) [13-15]. Nevertheless, supportive evidence for the application of VR in the rehabilitation of children with neurological disorders is still poor since the research is dominated by uncontrolled trials with only a small number of cases and case series [16].

**Figure 1:**
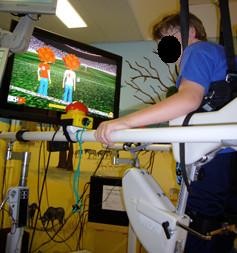
**Robotic assisted gait training (RAGT)**. Child on the pediatric Lokomat with the display presented to the subjects during VR Soccer condition.

Robotic-based technologies for gait rehabilitation, such as the driven gait orthosis Lokomat^® ^(Hocoma AG, Volketswil, Switzerland), offer highly standardized, repetitive gait training, relieve the therapists' physical strain of manually guiding training and allow objective measurements of performance and progress. On the other hand, it is difficult to estimate a patient's performance during robotic assisted gait training (RAGT) due to the loss of physical contact between therapist and patient [17,18]. Therefore, in RAGT it is essential that patients participate actively rather than just letting themselves "be walked". Combining the Lokomat with advanced VR technologies seems to be a promising option for rehabilitation therapy as it allows controlling and manipulating feedback parameters and thus leads to more challenging situations (Figure 1).

The present study was designed to systematically test the efficacy of combining the Lokomat with VR in children with central motor gait impairment and a healthy control group. We developed a motivational VR-based soccer scenario which provides interactive elements to engage patients during RAGT. Children's level of activity and participation during RAGT were quantified by weighted force measurements output by the Lokomat - the so-called biofeedback values [18]. The biofeedback values are weighted averages of the forces at the hip and knee joints, calculated for the stance and swing phase. In RAGT training without VR, therapists typically try to motivate the patient maximally to obtain higher force output in hip and knee muscles, which serves as an important training goal. In VR, the virtual scenario is supposed to adopt, at least partially, the motivational role of therapists. Therefore, in the present study we compared the immediate effect of different supportive conditions (therapist's instruction versus VR-based scenario) on motor output (biofeedback values). We hypothesize that the immediate motor output in all participants will be significantly higher during supportive conditions with VR compared to conditions without VR as a motivational factor.

## Methods

The study was approved by the local Ethics committee and brought into conformance with standards in the Declaration of Helsinki. Written informed consent was obtained from the legal guardians of all subjects before inclusion in the study. All measurements were conducted at the Rehabilitation Centre in Affoltern a. A. of the University Children's Hospital in Zurich, Switzerland.

### Participants

Current as well as former patients with neurological gait disorders of the Rehabilitation Centre Affoltern a. A. of the University Hospital Zurich were screened for eligibility. A total of 18 children took part in this study: Ten patients (four males, six females, mean age 14.2 years, SD 2.8 years) with different neurological gait disorders and eight healthy children (two males, six females, mean age 11.8 years, SD 3.3 years). Patients had an average weight of 46 kg (SD 12.1 kg) and an average height of 157 cm (SD 15 cm). Healthy control children had an average weight of 41.7 kg (SD 11.7 kg) and an average height of 149 cm (SD 13 cm), which did not differ significantly from the patient group. Demographic characterization of the participants is given in Table 1.

**Table 1:** Characteristics of participants with and without neurological gait disorders

**Subject No**.	Sex	Age (years)	Height (cm)	Weight (kg)	Lokomat's Legs K = Kids T = Teens	Diagnosis (GMFCS-Level)
VP_01	f	10.3	140	44.3	K	-
VP_02	m	13.5	154	40	T	-
VP_03	f	12.1	148	40.8	T	CP, diplegia (II)
VP_04	f	11.3	140	32.8	K	-
VP_05	m	8.4	127	25.0	K	CP, diplegia (II)
VP_06	m	15.11	168	47.8	T	CP, diplegia (II)
VP_07	f	16.10	178	60.8	T	Hip dysplasy
VP_08	f	9.3	137	32	K	-
VP_09	f	17.6	161	56.0	T	Cerebral hemorrhage
VP_10	f	15.4	168	50.1	T	MS
VP_11	f	10.10	140	34	K	-
VP_12	f	15.3	169	58.6	T	Encephalopathy
VP_13	m	16.8	158	46.7	T	CP, tetraplegia (III)
VP_14	m	8.11	143	33	K	-
VP_15	f	14.4	160	47.8	T	Symptomatic SCI
VP_16	f	17.2	168	53	T	-
VP_17	f	16.11	169	64.5	T	-
VP_18	m	13.1	139	27.0	K	CP, tetraplegia (II)

Abbreviations: CP = Cerebral Palsy; BS-CP = bilateral spastic cerebral palsy; MS = Multiple Sclerosis; SCI = Spinal cord injury

Inclusion criteria for all participants were: (1) aged 4-18 with a femur length between 21 and 47 cm (2) minimal voluntary control of their lower-extremity muscles to ensure that they had the ability to respond and adapt their walking and could follow different walking instructions (3) ability to signal pain, fear, discomfort and (4) willingness to meet the study requirements. One healthy control subject had to be excluded from the analysis due to data loss during recording.

### Virtual Environment System Setup

The VR setup was installed on the Lokomat, which consisted of a 42-inch flat screen and a 7.1 Dolby surround system. The graphic elements were programmed using the Ogre framework http://www.ogre3d.org. The sound output was rendered using the Fmod programmers API http://www.fmod.org and the graphics models were created in Maya http://www.adobe.com. The Lokomat system was used as a multimodal feedback system: the input device translated the subject's movements into movements of an avatar in the virtual environment (VE). Furthermore, the Lokomat was able to display that interactions with objects, such as a soccer ball, represented in the virtual environment with the purpose of providing haptic feedback to the subject. Koenig et al. [19] showed that the soccer simulation produces a physically realistic output force on ball contact.

The Biofeedback of the Lokomat gait orthosis is based on the interaction torques between the subject and the orthosis. For this reason, the hip and knee linear drives are equipped with force sensors, which measure the force that is required to keep the subject on the predefined gait trajectory [17]. For clinical use, the Lokomat is normally position-controlled with 100% guidance force. Changes in the participant's behavior are best detectable during this high stiffness, because small deviations lead to large counteracting forces. Additionally, we provided the possibility of free movements during a discrete event, i.e. for the leg swing during the kick of a soccer ball.

**Figure 2:**
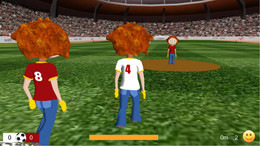
**Overview of the VR soccer scenario**. Displaying the VR soccer scenario with the two opponents in red (Image courtesy of Hocoma AG).

The soccer game made it possible for participants to kick a soccer ball in competition against two virtual opponents (Figure 2). One was waiting in front of the participant, who had to kick the ball past his opponent, otherwise he had to start from the last kick position. The second would approach from behind, taking over the soccer ball from the participant when he outpaced him. This second opponent was configured to walk faster and take over the ball from the participant if the exertion of the participant was weak and to walk slower when the subject participated actively. Within the VE, the position of the camera was slightly shifted to the right, providing an over-the-shoulder view. When the opponent was more than 1.68 m behind the avatar, the opponent was not visible on the VR screen. In our previous study [20], we were able to show increased mean biofeedback values for the time when the opponent was visible and decreased values for the time the opponent was not visible. Therefore, we assume that a constant competitive situation could serve as an additional motivational factor. Hence, in the current soccer implementation, the therapist was able to manipulate the opponent's speed offset and walking according to the skills of a participant.

### Procedures

**Figure 3:**
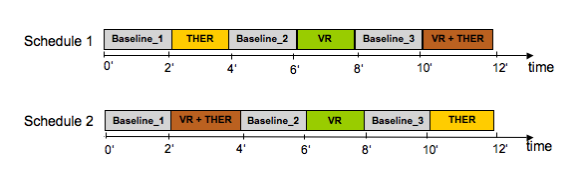
**The two different experimental schedule structures**. Showing the two different schematic time schedules for the study presented with all conditions. THER: Therapeutic instructions. VR: Virtual reality soccer scenario. VR + THER: Combination of VR and additional therapeutic instructions.

Participants were instructed to walk on the Lokomat under four randomly-presented conditions: (1) normal walking without supporting assistance from the therapist (BASELINE), (2) with therapists' standardized instructions to promote active participation (THER), (3) use of VR as a motivating tool to walk actively (VR), and (4) use of the VR tool combined with therapists' standardized instructions (VR + THER). The measured motor output was quantified by a weighted sum of interaction forces between patient and Lokomat which is computed for each swing and stance phase for both hip and knee joints [21]. The weighting functions were defined for each part of the gait cycle, such that the resulting biofeedback values increased for therapeutically desirable movements, e.g. knee flexion for early swing phase. All patients and healthy children were randomly assigned to two test schedules with balanced age distribution to avoid fatigue effect. After being fitted into the driven gait orthosis and before starting the first condition, children walked approximately five minutes in the Lokomat to familiarize themselves with the device. Each schedule began with and included in total three BASELINE measurements. Each walking condition lasted two minutes (Figure 3). During all conditions, children walked at their own comfortable speed (average for children's legs was 1.5 km/h, for teenager's legs 1.7 km/h) with 30% body weight support and foot-lifting straps, which assisted ankle dorsiflexion for adequate toe-clearance during the swing phase. All instructions given by the therapist were standardized for all conditions.

Participants' acceptance of Lokomat training with VR was assessed by a self-designed questionnaire for children. We asked the participants to rate the following points with regard to their experience with VR: their opinions about training with and without VR, the subjective value of the RAGT training in general and their own effort during the VR training. The questionnaire was presented as a visual analogue scale (VAS).

### Statistical Analysis

We recorded the four biofeedback values (bilateral hip and knee joints) during all conditions for the swing-phase only, because Lünenburger et al. [18] demonstrated that there was a high correlation between only the swing phase and the instructed activity, whereas correlation involving the stance phase was low and sometimes even negative. The biofeedback values are unit less, positive when the patient is actively participating and negative when the Lokomat carries the load of moving the patient on its predefined joint trajectory. To describe the individual overall walking performance under each condition, the mean of all four biofeedback values was calculated for each step. Thereafter, the mean of all biofeedback values during one condition was calculated. This provided one biofeedback value for each condition (BASELINE, THER, VR, VR + THER).

First, all data were examined for normality. The statistical analysis for the three baseline measurements in all subjects was calculated using repeated measures ANOVA. Motor output parameters were analyzed using a 2 × 4 mixed ANOVA with GROUP (patient versus healthy controls) as between-subjects factor and CONDITION (BASELINE, THER, VR, VR+THER) as within-subjects factor. A post hoc analysis was performed using a paired t-test for comparisons between conditions. In general, effects were considered meaningful when they fell below p < 0.05. We performed post hoc analysis using Bonferroni-Holm corrected t-tests for paired samples, applying the correction procedure that Holm [22] suggested. This procedure refers to a step-down method on the basis of classical Bonferroni-Holm correction for multiple comparisons. In the present article, the largest p value is adjusted according to the number of all tests (N), whereas the second most extreme p value is adjusted according to (N-1) tests, and so on. T values were reported as being significant only if the corresponding p value survived the correction procedure characterized by the initial p value of .05 and the number of tests. Statistical analysis was performed using the statistical software package SPSS 16 for Mac, release 16.0.1 software (SPSS Inc. 2007, http://www.spss.com).

## Results

The analysis of the three baseline (baseline_1, baseline_2, baseline_3) measurements revealed no significant main effect for the factor CONDITION (F = 1.779; p = 0.186) of the mean motor output. Therefore, the values for the three baseline conditions showed no fatigue effect and were allowed to be combined as mean total baseline. The mixed ANOVA revealed significant main effects for the factor CONDITIONS_(Baseline, VR, THER, VR+THER) _(F = 35,567; p < 0.001) and significant interaction CONDITIONS_(Baseline, VR, THER, VR + THER) _× GROUP_(patients, healthy controls) _(F = 4.268; p = 0.01). Tests of between-subjects effects showed no significant main effect for the GROUP_(patients, healthy controls) _(F = 0.3; p = 0.592). Contrasts of within-subjects revealed significant effects for the comparison baseline and therapist (F = 66.442; p < 0.001) and also for therapist and VR (F = 16.26; p = 0.001), but no statistical significant difference between VR and VR + THER (F = 0.682; p = 0.422). To break down the interaction, contrasts were performed comparing each condition across patients and healthy controls. These revealed significant interactions when comparing patients and healthy control values to baseline compared with THER (F = 6.571; p = 0.022) and to VR and VR + THER (F = 5.025; p = 0.041) but no significant effect to THER compared with VR (F = 0.663; p = 0.428).

**Figure 4:**
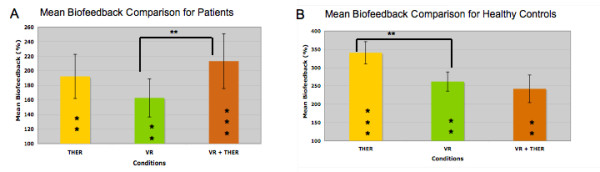
**Mean biofeedback improvement for patients and healthy control children**. A: Showing percent mean biofeedback improvements for patients in all conditions compared with baseline. ** within-group differences (p < 0.01); *** within-group differences (p = 0.001). B: Showing percent mean biofeedback improvements for healthy control children in all conditions compared with baseline. ** within-group differences (p < 0.01); *** within-group differences (p = 0.001).

Figure 4A and 4B show the mean motor output (measured as biofeedback values) improvement under all conditions compared with baseline for patients and healthy control subjects, respectively. Paired t-tests were analyzed separately for patients and healthy control subjects due to the main interaction effect (CONDITION × GROUP) of the ANOVA and were corrected for multiple comparisons (N = 6) as described in the methods section. For patients the biofeedback values revealed significant differences under all conditions compared with the total baseline condition (for THER t = -3.852, p = 0.005; for VR t = -3.496, p = 0.008 and for VR + THER t = -5.051, p = 0.001). Furthermore, significant results showed the comparison between VR and VR + THER (t = -3.548, p = 0.009), but not for both the comparison between therapist's instruction and VR (t = 1.688, p = 0.135) and for the combination of VR + THER with therapist's instructions alone (t = 1.245, p = 0.253). Similar results were found for healthy controls: paired t-tests revealed significant results for all supportive conditions compared with the total baseline condition (for THER t = -7.539, p < 0.001; for VR t = -4.634, p = 0.004, and for VR + THER t = -3.799, p = 0.009). Furthermore, significant differences were revealed by comparison of THER and VR (t = 4.034, p = 0.007) but not for comparison of THER and the combination of VR + THER (t = 2.552, p = 0.043).

**Figure 5:**
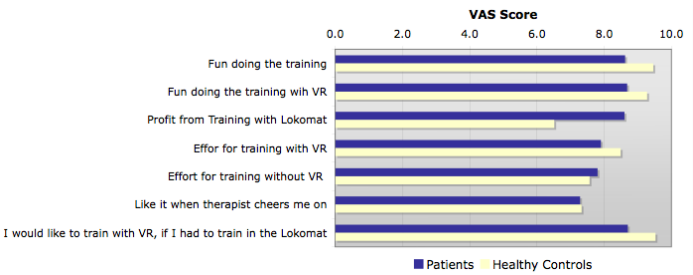
**Subjects opinion about RAGT with VR**. Motivation towards RAGT with and without VR was evaluated for all subjects using a self-designed written questionnaire presented as a VAS.

The analysis of the questionnaire (Figure 5) showed that all subjects had fun during the whole training session (mean for patients 8.7 points and for healthy control children 9.2 points, respectively). Healthy control subjects achieved slightly higher scores most of the time on the VAS than patients except for one question concerning profit from the Lokomat training. This may be because this question is aimed at RAGT with patients. It is difficult for healthy children to visualize the benefit of rehabilitation training. Patients and healthy control children reported somewhat less inspiration by therapists than during VR.

## Discussion and Conclusions

The aim of this study was to compare the effect of different supportive strategies during RAGT on the degree of active participation in children. This work investigated differences in therapy conditions on a single day and showed active participation during a short time period of two minutes. Within this period, we showed that VR has the same immediate effect on motor output as therapist instructions in subjects with neurological gait disorders. Most importantly, the study revealed that both children with and without neurological disorders achieved significantly higher motor output during all supportive conditions as compared to walking without any motivational assistance. In other words, active participation was increased either by verbal encouragement given by a physical therapist (THER), by a VR soccer scenario or by the combination of both (VR + THER). It is not yet known whether such enhanced active performance can also be maintained over longer time periods or during a whole training session and whether this leads to a more effective rehabilitation process for patients. Furthermore, as there was no significant difference between the motor output measures and the three baseline measurements (walking without motivational assistance), it might be inferred that, with regard to the degree of active participation, the walking at the beginning of a therapeutic session is comparable to that at the end and shows no general fatigue effect. Although fatigue was not systematically verified during the training session, it might be interesting to include a fatigue score in further research.

It has been proposed that active training is more effective than passive training for motor learning and cortical reorganization [9]. Important findings in stroke patients suggest that simply moving or passively exercising the impaired limb does not lead to maximum recovery. Furthermore, it has become apparent that new motor skills, enriched, highly functional and task-oriented practice environments and primarily motivating tasks which increase engagement are necessary for motor re-learning and recovery after stroke [23]. Although children with CP might be substantially different in motor learning than those having experienced stroke or spinal cord injury, in cases in which the patients did have an intact and normally functioning nervous system prior to injury, it has been shown that activity, task-specificity and goal-orientedness are also crucial aspects in treatment of children with CP [24,25].

Therefore, in RAGT it is essential that patients participate actively instead of just letting themselves "be walked". The patient's performance during the RAGT is difficult to estimate due to the loss of physical contact between therapist and patient [17,18]. With the advanced biofeedback facility integrated in the Lokomat system used for the present study, we were able to record force interaction between the patient and the Lokomat and, on the basis of this data, to estimate the subject's performance. Although Lünenburger et al. [18] could demonstrate that biofeedback values are useful for evaluating and assessing the walking performance of subjects during Lokomat training, only the values for swing-phase correlated highly with the instructed activity, whereas the correlation of the stance-phase was less and sometimes even inversely correlated. Therefore, we recorded the four biofeedback values (bilateral hip and knee joints) during all conditions for the swing-phase only.

As outlined in the introduction, patient motivation plays a crucial role in determining therapy outcome, especially in the field of pediatric rehabilitation. The RAGT sessions, which consist of standardized monotonous walking for 30-45 minutes, are usually rather boring for children and can even be inconvenient. Hence, pediatric rehabilitation centers using RAGT try to boost patient motivation by showing DVDs or playing music. Such strategies, however, may distract children from the actual therapy, causing them to become completely passive in the Lokomat. VR techniques make it the possible to directly interlink the patients' motor performances during the gait training with actions in a computer-game-like virtual world. VR games adequately adapted to children's needs provide motivation and yet keep the focus on the actual gait training. Furthermore, the VR soccer scenario used is adaptable to children's individual skill levels and adjusts interactive elements to maximize motivation. In the current VR soccer implementation, the therapist could manipulate the opponent's speed offset and walking speed according to the skills of the participants. Assuming that a constant competitive situation could serve as a motivational factor, we included two different opponents in the present VR, one represents the first line of defense, over which the participant must kick the ball. The second approaches from behind and is able to take over the soccer ball from the avatar when he is in front.

In this study, we investigated the effect of adopting a VR scenario during RAGT based on the individual's level of active participation and compared this to a regular training session involving therapist encouragement and motivation. It should be pointed out, however, that the social interaction between a therapist and participant undoubtedly plays a crucial role, especially for patients. Thus, the use of VR during rehabilitation therapy should not replace the physical therapist, but rather provide an additional means of enhancing training efficiency.

Children with neurological disorders as well as healthy controls achieved higher active participation levels not only with therapist encouragement but also with a VR soccer scenario during RAGT. Based on our clinical experience, the measurements gathered indicate that higher motivation and focused attention during RAGT have a positive influence on children's motor output, which in turn might lead to enhanced motor learning. Further research is required in this area.

Given that the four supportive conditions varied in patients and healthy control children, we will compare and discuss these conditions for the two groups separately. Besides the fact that the mean motor output for patients revealed significant differences under all conditions involving motivational assistance compared with the normal walking condition, we also found significant differences between VR and VR combined with therapist instruction. All other comparisons of the supportive conditions exposed no significant differences.

It should be noted that the therapist's behavior during the two minutes of the "therapist-only" condition of the present study is not likely to be representative of normal behavior during a standard RAGT session of 30-45 minutes. In fact, motivating children during an entire training session is a very difficult and exhausting task and requires a great amount of engagement, creativity or even imagination. The use of a VR environment in RAGT, on the other hand, has the potential to constantly enhance and adapt training motivation and therefore increase active participation and training outcome. Moreover, VR may also be viewed as an additional medium used by the therapist to convey motivation and encouragement, e.g. by cheering when the patients' performance was particularly good or by encouraging the patient when something special must be achieved in the VR environment. This idea is in accordance with the fact that the combined condition VR+THER was significantly better than VR alone.

While mean active participation during baseline condition was similar for both groups, healthy control children achieved higher mean biofeedback values than patients for the condition therapist and the condition VR, but the difference was not significant. Furthermore, in healthy control children, there were significant differences between comparison therapist's instruction and VR values.

One explanation for the difference between patients and healthy children may be found in the safety system of the Lokomat. The device has built-in force monitoring which stops the robotic drives if participants provide too much force input. These technical limitations influenced the measurements, primarily those of the healthy children because healthy children have more power than patients and therefore occasionally activated the safety mechanism. Hence, some conditions may be slightly underestimated in terms of motor output values. On several occasions, the force exerted under VR and VR plus therapist's instruction conditions triggered the Lokomat's safety mechanism. This led to frustration, which in turn caused the healthy children to reduce their force and therefore produce lower motor output values than would otherwise have been possible during the affected conditions. This may explain decreased results during VR and VR+THER conditions in healthy control subjects.

In order to gain knowledge about the patient's perspective regarding the motivational properties of the soccer scenario used during RAGT, participants were asked to complete a self-designed motivation questionnaire. Overall the answers submitted indicated that all participants had fun during RAGT, were highly motivated and had done their best.

We are aware of potential shortcomings in our study, one of which might be the choice of the tested schedule order. Although, attempts were made to alter the order of the conditions, the VR alone condition was always placed in the middle of the session. As a result, subjects always had some practice with the Lokomat system before participating in the VR condition, which might have improved their performance. Secondly, the patient group may be biased due to previous experiences with training on the Lokomat and also with VR scenarios. However, the positive results obtained with the VR soccer condition seem to indicate the motivational aspect of VR games. Other limitations of this study are the small sample size of the groups as well as the heterogeneous abilities of the patients. Therefore, it may be difficult to make generalizations regarding the benefit of using VR as a motivational tool in RAGT with other patient populations.

VR in rehabilitation has become a promising and useful adjunct to traditional therapy by providing objective quantification of the training process as well as safe environments which motivate children to exercise [16,26]. The VR scenario presented has the potential to achieve higher motor outputs in children with neurological disorders as well as in healthy controls. Our observations support the idea that VR might be a promising supplement for RAGT in pediatric rehabilitation. However, further research and development is necessary in order to optimize such VR systems as a motivational tool and to investigate their clinical effectiveness in the rehabilitation process. Follow-up studies are needed in order to determine if the increase in active participation caused by patient cooperative strategies like VR leads to better clinical outcome. In addition, emphasis should be placed on the development of engaging and immersive game designs which allow for human gait variability and performance levels. These variables must be optimized in order to keep children attentive during consecutive training sessions of 30-40 minutes.

In summary, the VR scenario used here has an immediate effect on motor output (biofeedback values) similar to one resulting from verbal instructions by a therapist. Therefore, VR represents a valuable tool to keep patients and healthy control children participating actively during RAGT.

## Abbreviations

VR: Virtual reality; CP: Cerebral palsy; RAGT: Robotic assisted gait training; THER: Therapeutic instructions; VE: Virtual environment; VAS: Visual analogue scale; MMC: Meningomyelocele, a form of spina bifida; MS: Multiple sclerosis; SCI: Spinal cord injury.

## Competing interests

LZ and LL were employed by Hocoma AG, Volketswil, Switzerland, the producer of the Pediatric Lokomat. AMH was reimbursed by the Hocoma AG, for attending two conferences as an invited speaker and also received a fee for speaking at one conference.

## Authors' contributions

KB was involved in developing the study design, acquiring data, completing data analysis and drafting the manuscript. TS developed the study design, recruited subjects and performed data acquisition. AK, LZ, LL and RR developed the software and edited the manuscript. SK, AMH and LJ assisted with data interpretation as well as in revising the manuscript. All authors read and approved the final manuscript.
